# Protection from malaria infection using liver-targeted siRNA

**DOI:** 10.1016/j.omtm.2025.101516

**Published:** 2025-06-18

**Authors:** R.W.J. Steel, A. Schepis, T. Nguyen, S. Milstein, K. Yucius, H.C. Tu, E. Fishilevich, P. Haslett, S.H.I. Kappe

**Affiliations:** 1 Center for Global Infectious Disease Research, Seattle Children’s Research Institute, 307 Westlake Avenue North, Suite 500, Seattle, WA 98109, USA; 2 Alnylam Pharmaceuticals, 675 West Kendall St, Cambridge, MA 02142, USA; 3 Department of Pediatrics, University of Washington, Seattle, WA, USA

**Keywords:** malaria, malaria prevention, *Plasmodium*, siRNA, liver infection

## Abstract

Malaria-causing *Plasmodium* parasites infect the liver and undergo obligate and asymptomatic replication in hepatocytes prior to infection of the blood. As such, preventing liver infection will prevent blood infection, thereby prevent malaria disease, and the spread of parasites by mosquitoes. The tetraspanin CD81 is expressed on the hepatocyte plasma membrane and is a critical entry receptor for sporozoites of the major human parasite *Plasmodium falciparum.* Yet, the importance of this molecule has not been effectively exploited for malaria prevention. RNA interference (RNAi) is a clinically proven technology for the silencing of human disease-related genes and may thus represent a promising avenue for malaria prevention. Here, we report the application of CD81-targeted, N-acetylgalactosamine (GalNAc)-conjugated small interfering RNA (siRNA) to efficiently silence both mouse and human hepatic CD81 *in vivo*. Using a mouse model of malaria, we show that CD81 GalNAc-siRNA blocked parasite liver infection in a dose-dependent manner and prevented the onset of blood stage infection. We further provide evidence that this approach reduced *P. falciparum* infection using human liver-chimeric mice. The data suggest siRNA as a promising host-based approach to prevent malaria infection.

## Graphical abstract

**Figure:**
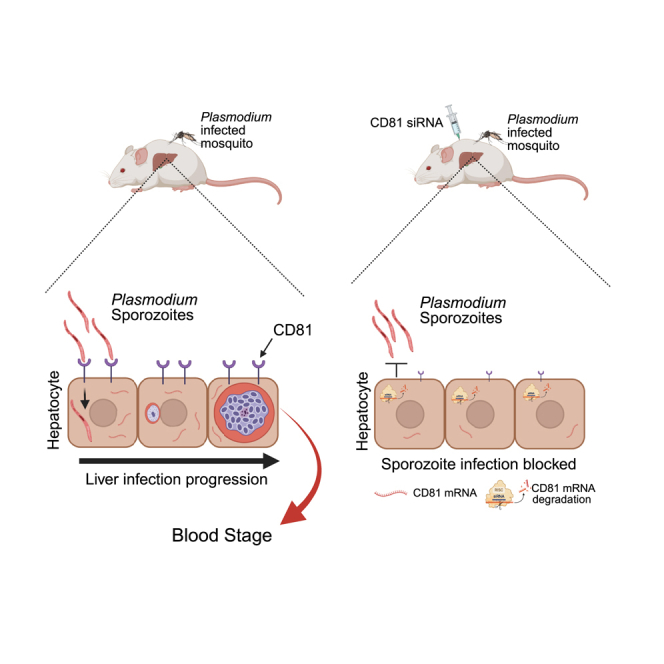

## Teaser

Malaria parasites first infect the liver and then infect red blood cells, which causes disease. Kappe and colleagues use an RNAi approach to target the hepatocyte entry receptor CD81. They show that *in vivo* liver knockdown of CD81 blocks parasite liver infection, preventing the occurrence of symptomatic blood stage infection.

## Introduction

Malaria, caused by *Plasmodium* parasites, is a global health problem, leading to disease in hundreds of millions of people annually.^1^ The most severe form of malaria in humans is caused by *Plasmodium falciparum*, which is responsible for the more than 600,000 deaths annually.^1^ Infection with *Plasmodium* parasites starts when sporozoite stages are injected into the host skin by a mosquito bite.^2^ The sporozoites then enter the vasculature and are transported to the liver, where they infect the hepatocytes. Within their intra-hepatocytic niche, parasites undergo exo-erythrocytic schizogony to generate tens of thousands of infectious merozoites, which upon release, infect red blood cells.^3^ Blood infection is responsible for all malaria symptoms and enables the completion of the parasite life cycle by sexual stage formation and onward transmission of these parasite stages to mosquitoes.^4^ Thus, targeting the pre-erythrocytic phases of infection, for example, by blocking sporozoite invasion of the hepatocytes, represents an attractive point of intervention, since a relatively small number of asymptomatic parasites can be targeted before disease onset. Two subunit vaccines based on the circumsporozoite surface protein (CSP) have been approved by the World Health Organization for use in Africa, based on their modest efficacy in preventing clinical malaria.^5^^,^^6^ In addition, monoclonal antibodies against CSP have recently shown good efficacy in passive immunization studies.^7^ However, host-based preventive therapeutics for the prevention of malaria infection have not been well explored.

Sporozoite invasion requires interaction between the parasite and host plasma membrane proteins.^2^ Relatively little is known about the molecular interactions of sporozoites with hepatocytes that are needed for successful infection, although a few key interactions have been revealed.^8^^,^^9^^,^^10^^,^^11^^,^^12^^,^^13^ The secreted sporozoite proteins P36 and P52 form an invasion complex to facilitate hepatocyte infection,^14^ with the P36 partner appearing to determine host receptor usage by different *Plasmodium* species.^8^ An important host receptor is the membrane tetraspanin CD81, which is critical for *P. falciparum* hepatocyte entry and entry by rodent-infective *Plasmodium yoelii* parasites*,* while scavenger receptor BI (SR-BI) is the critical host determinant for *Plasmodium vivax*.^8^^,^^9^^,^^10^^,^^15^ The requirement for these receptors at the entry step of hepatocyte infection by sporozoites identifies them as high value host factors for intervention, but to date, no clinical molecules capable of interrupting these critical host-pathogen interactions to prevent infection have been identified.

RNA interference (RNAi) is a naturally occurring pathway to regulate gene expression by targeting mRNA degradation in a target sequence-specific manner.^16^ Synthetically produced, chemically modified, small interfering RNA (siRNA) is an emerging therapeutic modality that enables the downregulation of intracellular target mRNA in a highly potent and durable manner.^17^ Delivery of siRNA to the hepatocytes can be achieved by conjugating siRNA molecules to trivalent N-acetylgalactosamine (GalNAc), a ligand with high affinity to the asialoglycoprotein receptor (ASGPR) in hepatocytes.^18^ One notable hallmark of the GalNAc-siRNA pharmacodynamics is its long duration of action enabling stable knockdown of the target gene with quarterly or bi-annual dosing regiments in human, mainly due to advanced chemical modifications that increased the metabolic stability of the siRNAs in the cells.^18^^,^^19^^,^^20^ To date, there are four Food and Drug Administration (FDA)-approved GalNAc-siRNA therapeutics and multiple active clinical programs in progress.^17^^,^^20^ Here we report the application of a GalNAc-conjugated siRNA to efficiently silence both mouse and human hepatic CD81 *in vivo*. We demonstrate the potential utility of this approach for the prevention of infection in the rodent malaria model *P. yoelii* and using a humanized mouse model for prevention of human hepatocyte infection with *P. falciparum*.

## Results

### *CD81* target design and validation

**Figure 1:**
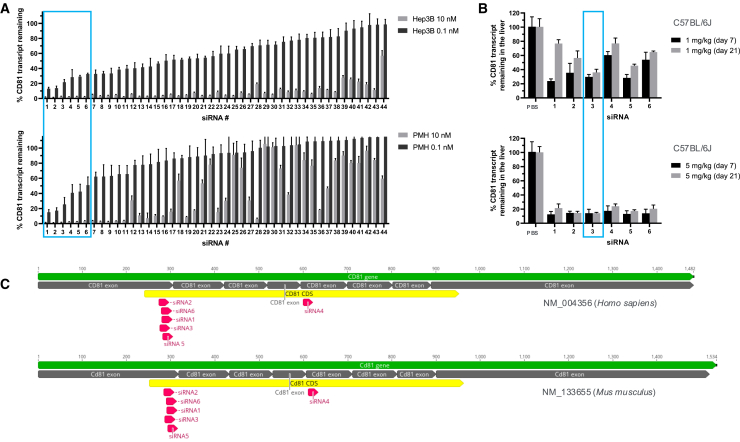
*In vitro* and *in vivo* activity of CD81-targeting siRNAs (A) *In vitro* activity, %*CD81* transcript remaining in Hep3B (top) and primary mouse hepatocytes (PMH, bottom) 24 h after transfection at 10 and 0.1 nM doses measured by qPCR. (B) *In vivo* activity of six most potent siRNAs from *in vitro* screen, %*CD81* transcript remaining in the livers of C57BL/6J mice 7 days and 21 days after subcutaneous siRNA doses of 1 (top) and 5 mg/kg (bottom) measured by qPCR. (C) Human/mouse cross-reactive siRNAs 1–6 mapped onto human and mouse RefSeq CD81 transcripts.

To identify siRNA sequences capable of efficiently silencing both human and mouse *CD81* in hepatocytes, a total of 44 siRNAs predicted to have high potency and cross-reactivity with human (NM_004356) and mouse (NM_133655) *CD81* nucleotide sequences were synthesized as GalNAc-siRNA conjugates and screened for their RNA transcript suppression efficacy at 10 and 0.1 nanomolar (nM), 24 h after their addition to human Hep3B and mouse C57Bl/6 primary hepatocyte in cell cultures (Figure 1A and Table S1). The majority of siRNAs achieved greater than 80% knockdown of human CD81, with approximately half also achieving this threshold in mouse primary hepatocytes. The six most potent siRNAs at the 0.1 nM dose were further screened in C57Bl/6 mice at single subcutaneous doses of 5 and 1 mg/kg (Figure 1B and Table S1). Liver CD81 mRNA levels were analyzed at seven and 21 days after treatment by qPCR, with mRNA extracted from whole livers. We observed strong *in vivo* knockdown at 5 mg/kg for all six siRNA molecules tested, and the effect lasted at least 21 days post-siRNA dosing (Figure 1B and Table S1). Treatment of mice at 1 mg/kg siRNA revealed differentiation of the potency between siRNAs—five of the six siRNAs showed greater than 60% transcript suppression at 7 days post-siRNA dosing at 1 mg/kg (Figure 1B). These most potent siRNAs spanned an overlapping region at exon 1/2 junction (Figure 1C), suggesting a potential hotspot region for siRNA targeting in the CD81 transcript. Among these siRNA molecules, siRNA 3 maintained >60% and >80% transcript suppression at 1 and 5 mg/kg, respectively, through day 21 post-dosing. On this basis, siRNA 3 was selected for use in *Plasmodium* spp. infection studies.

### Silencing of liver CD81 protects against *P. yoelii* and *P. falciparum* infection *in vivo*

**Figure 2:**
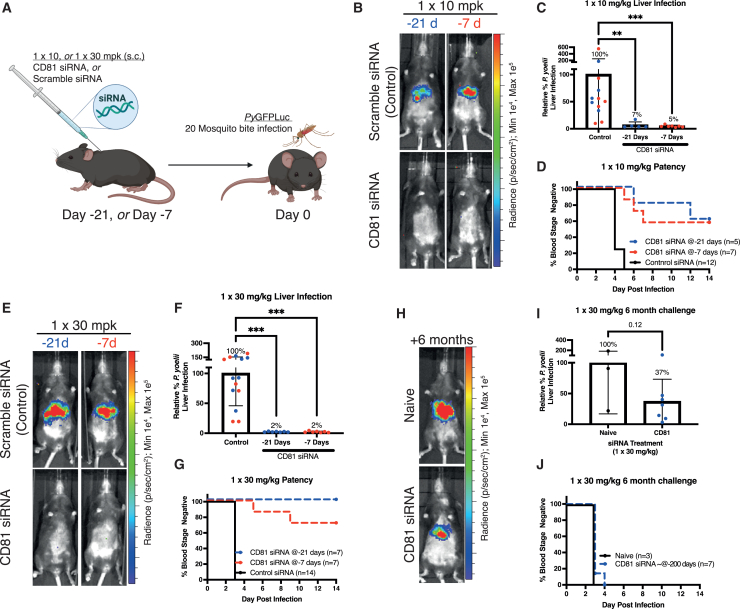
Suppression of hepatic mouse CD81 by GalNAc-siRNA confers protection against *P. yoelii* sporozoite infection *in vivo* (A) Experimental design for (B–G). Female C57Bl/6 mice received a single subcutaneous injection of CD81-targeting or CD81-scramble (control) siRNA 21 or 7 days before infection by the bites of 20 mosquitoes infected with PyGFPLuc for 15 min. (B) Representative *Py*GFPLuc liver infection level measured by IVIS at 44 hpi in mice treated with 1 × 10 mg/kg s.c. of either siRNA at the indicated days before infection. (C) The *Py*GFPLuc liver infection from all mice treated with 1 × 10 mg/kg s.c. siRNA is shown relative to control and normalized infection percentage shown above bars. (D) Time to patency for all mice that received 1 × 10 mg/kg s.c. siRNA as assessed using Giemsa-stained thin blood smears. (E) Representative *Py*GFPLuc liver infection level measured by IVIS at 44 hpi in mice treated with 1 × 30 mg/kg s.c. of either siRNA at the indicated days before infection. (F) The *Py*GFPLuc liver infection from all mice treated with 1 × 30 mg/kg s.c. siRNA is shown relative to control; normalized infection percentage shown above bars. (G) Time to patency for all mice that received 1 × 30 mg/kg s.c. siRNA as assessed using Giemsa-stained thin blood smears. (H) Representative *Py*GFPLuc liver infection level measured by IVIS 44 hpi in naive mice and those undergoing a second challenge ∼6 months (200 days) after 1 × 30 mg/kg s.c. CD81-targeting siRNA. (I) The *Py*GFPLuc liver infection from naive and CD81-targeting siRNA mice during their second challenge relative to control; normalized infection percentage shown above bars. (J) Time to patency for naive and CD81-targeting siRNA mice during their second challenge as assessed using Giemsa-stained thin blood smears. Data represent the mean ± SD of individual mice with parametric analysis by one-way ANOVA with Holm-Sidak’s multiple comparison test (in F) or t test (in I), or non-parametric analysis by Kruskal Wallis test with Dunn’s multiple comparisons test (in C). In all plots, blue data points represent mice treated 21 days before infection and red data points represent mice treated 7 days before infection. *∗∗p* < 0.01 and *∗∗∗p* < 0.001; mpk = mg/kg, milligrams per kilogram; hpi, hours post infection; s.c., subcutaneous.

Having identified siRNA 3 as a potent GalNAc-siRNA molecule targeting both mouse and human CD81, we next sought to determine its protective efficacy against *Plasmodium* spp. infection. We used the *P. yoelii* rodent malaria parasite that depends on CD81 for infection of hepatocytes, similar to *P. falciparum.*^10^ We first undertook a single dose escalation study using CD81-targeting siRNA 3 or its scrambled CD81 control siRNA molecule in C57Bl/6 mice. Mice were subcutaneously injected with GalNAc-siRNAs 21 days or 7 days before infection (BI) with *P. yoelii* sporozoites expressing luciferase, (*Py*GFPLuc)^21^ by mosquito bite (Figure 2A). Protection against liver infection was measured by bioluminescent imaging at the peak of liver stage parasite development, at 2 days post-infection (PI). Mice were also monitored for the emergence of parasites in the blood (a stringent and sensitive measurement for complete protection against pre-erythrocytic infection) starting at 3 days PI. Mice treated with 10 mg/kg siRNA 3 at 21 or 7 days BI displayed more than 90% reduction in liver infection compared with the scrambled siRNA-treated mice (Figures 2B and 2C). Moreover, some mice were completely protected as indicated by the absence of blood stage infection, with the 10 mg/kg siRNA 3 at day BI group showing the highest level of protection (60%). The remaining mice displayed a delay in blood stage emergence compared to the control siRNA-treated mice (Figure 2D).

Increasing the dose of siRNA from 3 to 30 mg/kg showed a more pronounced effect on liver infection, with decreases in liver stage parasite burden up to 98% when siRNA was administrated either at 21 days or 7 days BI (Figures 2E and 2F). When a 30 mg/mL dose was given at 7 days BI, we did not detect blood stage emergence for most of the mice treated with siRNA 3 (Figure 2G), and the mice which did show blood stage infection showed a delay in blood stage emergence (Figure 2G). Remarkably, all mice which were given a 30 mg/mL dose at 21 days BI were completely protected from the onset of blood stage infection (Figure 2G). Six months after siRNA treatment, mice that had been given a 30 mg/mL dose were challenged for a second time. Liver infection was reduced approximately 60% compared to the scrambled siRNA (Figures 2H and 2I). Of note, while durable knockdowns have been shown in clinical setting,^19^^,^^22^^,^^23^ we cannot exclude that the liver infection reduction was instead caused by the immune response to sporozoites generated by the first challenge. Moreover, the liver infection reduction for the second challenge did not prevent the emergence of blood stage infection (Figure 2J).

**Figure 3:**
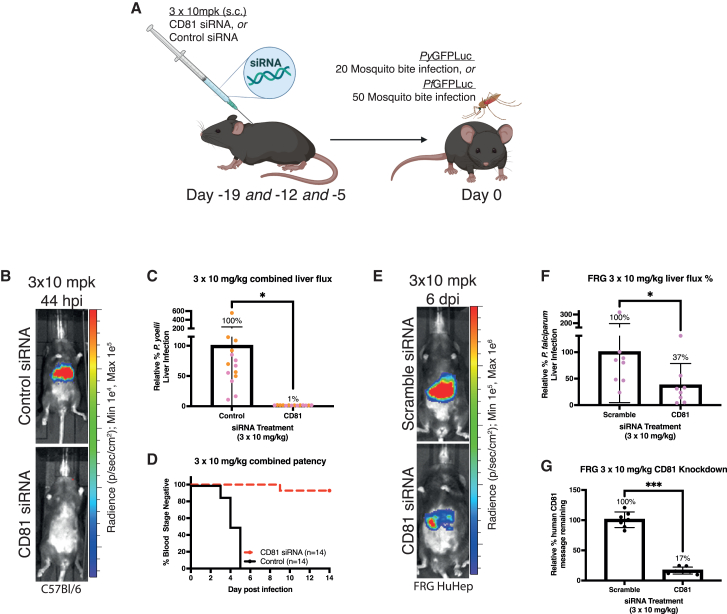
Suppression of hepatic human CD81 by GalNAc-siRNA achieves *in vivo* protection against *P. yoelii* and *P. falciparum* (A) Experimental design for (B–G). Mice received three subcutaneous injections of the CD81-targeting or control (scramble) siRNA at 10 mg/kg on days 19, 12, and 5 before infection with PyGFPLuc or PfGFPLuc for C57Bl/6 or FRG-HuHep mice, respectively. (B) Representative *Py*GFPLuc liver infection level measured by IVIS at 44 hpi in mice treated with 3 × 10 mg/kg s.c. siRNA before infection. (C) The *Py*GFPLuc liver infection from all mice treated with 3 × 10 mg/kg s.c. siRNA is shown relative to control; normalized infection percentage shown above bars. (D) Time to patency for all mice that received 3 × 10 mg/kg s.c. siRNA as assessed using Giemsa-stained thin blood smears. (E) Representative PfGFPLuc liver infection level measured by IVIS at 6 dpi in FRG-HuHep mice; data from all mice are shown relative to control in (F). (F) The PfGFPLuc liver infection from all mice treated with 3 x 10 mg/kg s.c. (G) Measurement of human CD81 mRNA knockdown by 3 × 10 mg/kg in FRG-HuHep mice. Data represent the mean ± SD of individual mice with parametric analysis by one-way ANOVA with Holm-Sidak’s multiple comparison test (in F) or t test (in I), or non-parametric analysis by Kruskal Wallis test with Dunn’s multiple comparisons test (in C). *∗∗p* < 0.01 and *∗∗∗p* < 0.001; mpk = mg/kg, milligrams per kilogram; hpi, hours post infection; s.c., subcutaneous.

Next, we tested a fractional approach with three doses of siRNA 3 given at 19, 12, and 5 days BI (Figure 3A). We observed that siRNA 3 × 3 fractional doses caused a near complete reduction of liver infection (∼99%), compared to the control siRNA, and prevented the emergence of blood stage infection in nearly all mice (Figures 3B–3D).

Finally, we investigated whether siRNA could also prevent liver infection by the human-infective malaria parasites using the fumarylacetoacetate hydrolase (FAH)-, Rag2-, and the common gamma chain of the interleukin receptor knockout (FRG) HuHep mouse model, in which the mouse liver is repopulated with human hepatocytes. These mice are susceptible to *P. falciparum* liver infection.^24^ We used a *P. falciparum* strain that expresses luciferase for sporozoite challenge experiments by mosquito bite.^25^ The fractional dose protocol with three doses of siRNA 3 given at 19, 12, and 5 days BI reduced human hepatic CD81 expression in humanized FRG-HuHep mouse livers by approximately 83%. This resulted in an approximately 63% reduction of *P. falciparum* liver infection compared to the control siRNA group (Figures 3I–3K).

## Discussion

*P*. *falciparum* malaria is a devastating disease that kills hundreds of thousands of people every year in malaria-endemic regions of world^1^ and also puts tens of millions of travellers to malaria-endemic areas at risk of infection each year. Malaria is currently controlled by targeting the mosquito vectors, preventing mosquito bites, and by drug treatment. However, the emergence of *P. falciparum* strains resistant to drugs such as artemisinin challenges sustainable malaria treatment efforts.^26^ Recently approved vaccines for *P. falciparum* malaria are expected to reduce the clinical disease burden of malaria but are not expected to eliminate or eradicate malaria.^27^^,^^28^ Thus, the discovery and development of new malaria therapeutics is required and there is an urgent need for interventions that prevent malaria parasite infection.

In this study, we tested siRNAs as preventative therapeutic to block *Plasmodium* infection of the liver. SiRNAs have been proven clinically successful against certain diseases, with durable effect.^17^^,^^19^^,^^20^ As proof of concept, we targeted the hepatocyte tetraspanin CD81, a critical host factor for hepatocyte infection by *P. falciparum* sporozoites, with a specific siRNA.^15^^,^^29^ Using the rodent malaria parasite *P. yoelii*, which also requires CD81 for hepatocyte infection,^8^^,^^30^ we showed that a single high dose or a fractional multi-dose siRNA injection was highly effective in preventing liver infection and, thereby, the parasites transition to pathogenic blood stage infection. Moreover, we showed that this approach reduces liver infection by the *P. falciparum* in a liver-humanized mouse model. Although the effectiveness of the siRNA to reduce liver infection persisted to a significant degree over time, the application and dose regimens used herein were not sufficient to durably prevent blood stage infection. However, knockdown efficiency can be different in different models, species, and experimental settings. For instance, we selected siRNA that could target both murine and human CD81. While our study provides a proof of concept, future efforts should focus on improving siRNA potency and dosing regimens that would specifically prevent human malaria parasite liver infection. Targeting a host factor for prevention of pathogen infection raises concerns about detrimental effects on the host. We did not observe any overt negative health effects on CD81 siRNA-treated mice in our experiments. Furthermore, a human case report of homozygous loss of function of CD81 was described; the major clinical manifestation was humoral immune deficiency, which was thought to be driven by the lack of CD81 expression in the B cells.^31^ Unlike small molecule or antibody-based approaches, GalNAc-conjugated siRNA minimizes the risk of affecting CD81 expression in B cells due to the specificity of ASGPR expression in hepatocytes. Experiments with GalNAc-conjugated siRNA targeting CD45, which is expressed in nucleated hematopoietic cells, including B cells, show that GalNAc mediates no siRNA activity in B cells (Figures S1A and S1B). Importantly, there were no reported liver-related consequences in the aforementioned case report.^31^ In addition, there were no reported liver pathologies in a CD81 knockout mouse model.^32^ Collectively, those studies support the safety of a hepatocyte-specific CD81 targeting strategy.^31^^,^^32^

Additional siRNA targets for prevention of infection by other human-infective malaria parasite species should be investigated, such as scavenger receptor B1, a critical hepatocyte entry factor for *P. vivax*.^8^ This approach might provide significant clinical benefit, even when only partially effective in preventing primary infection by this parasite, as it was shown that partial reduction of liver stage parasite burden during primary infection prevented a high proportion of relapsing infection in a liver humanized mouse mode.^33^ Taken together, our results provide unprecedented proof-of-concept evidence that siRNA-based therapeutics are promising tools for the prevention of malaria.

## Materials and methods

### Ethics statement

All animal experiments conducted at Seattle Children’s Research Institute (previously Centre for Infectious Research) were conducted in accordance with approved protocols reviewed by the Seattle Children’s Research Institute Institutional Animal Care and Use Committee (IACUC) under approval no. IACUC00513.

Animal experiments at Alnylam Pharmaceuticals were conducted in accordance with approved protocols, reviewed by the Alnylam Pharmaceuticals IACUC. These protocols are reviewed annually and revised triennially by the appropriate Principal Investigator and IACUC. Alnylam’s IACUC adheres to the standards set by the Association for Assessment and Accreditation of Laboratory Animal Care and the *Guide for the Care and Use of Laboratory Animals: Eighth Edition*.

### Mice

Female Swiss Webster mice (Envigo Laboratories) were used for the maintenance of the *P. yoelii* life cycle required to produce infected mosquitoes. For analysis of CD81 targeting siRNA, female C57Bl/6 mice (6–8 weeks olds) (Jackson Laboratories) and approximately 3-month-old FRG-HuHep mice (Yecuris) were used for *P. yoelii* and *P. falciparum* infection, respectively. All mice were maintained under standard pathogen free conditions at the Center for Global Infectious Disease Research, Seattle Children’s Research Institute.

### Design and testing of siRNA molecules

GalNAc-conjugated siRNA cross-reactive against human (NM_004356) and rodent (NM_133655) CD81 transcripts and rodent tyrosine phosphatase receptor type C (CD45) (NM_001111316) were designed using Alnylam proprietary algorithms. As described earlier,^18^ siRNAs were synthesized as fully modified sense and antisense strands containing 2′-Fl and 2′-O-Me modifications. For The CD45 experiments, C12-200 cationic lipid nanoparticles (LNPs) were formulated with siRNA as previously reported.^34^^,^^35^ siRNAs were administered to mice by subcutaneous (s.c.) injection at the scruff of the neck. LNPs were administered by an intravenous bolus injection at 1 mg/kg and siRNAs were administered by subcutaneous injection at 30 mg/kg. After 7 days for GalNAc conjugates and 3 days for LNPs, animals were euthanized and tissues harvested and processed as previously described.^35^

### Cell culture and transfections

Primary mouse hepatocytes (PMH) (GIBCO) and human Hep3b (ATCC) cells were transfected by adding 4.9 μL of Opti-MEM plus 0.1 μL of Lipofectamine RNAiMax per well (Invitrogen, Carlsbad, CA, cat no. 13778-150) to 5 μL of siRNA per well into a 384-well plate and incubated at room temperature for 15 min. William’s E Medium (40 mL) (Life Tech) or Eagle’s minimal essential medium (ATCC) containing ∼5 × 10^3^ cells were then added to the siRNA mixture. Cells were incubated for 24 h prior to RNA purification. Single dose experiments were performed at 10 and 0.1 nM siRNA concentrations. siRNA targeting luciferase was used as a negative control.

### Total RNA isolation using Dynabeads mRNA isolation kit

Total RNA was isolated using an automated protocol on a BioTek-EL406 platform using Dynabeads mRNA Isolation Kit (Invitrogen, cat no. 61012). Briefly, 50 μL of lysis/binding buffer and 25 μL of lysis buffer containing 3 mL of magnetic beads were added to the plate with cells. Plates were incubated on an electromagnetic shaker for 10 min at room temperature, and then magnetic beads were captured and the supernatant was removed. Bead-bound RNA was then washed 2 times with 150 μL Wash Buffer A and once with Wash Buffer B. Beads were then washed with 150 mL Elution Buffer, re-captured, and the supernatant was removed.

### cDNA synthesis

cDNA was synthesizes using ABI High-Capacity cDNA Reverse Transcription Kit (Applied Biosystems, Foster City, CA, cat no. 4368813) as follows. A master mix (10 μL) containing 1 μL 10× buffer, 0.4 μL 25× dNTPs, 1 μL 10× random primers, 0.5 μL reverse transcriptase, 0.5 μL RNase inhibitor, and 6.6 μL of H_2_O per reaction was added to RNA isolated previously. Plates were sealed, mixed, and incubated on an electromagnetic shaker for 10 min at room temperature, followed by 2 h at 37°C.

### Real-time PCR

cDNA (2 μL) were added to a master mix containing 0.5 μL of Mouse GAPDH TaqMan Probe (Applied Biosystems, 4352339E) or 0.5 μL of human GAPDH TaqMan Probe (4326317E), 0.5 μL CD81 mouse probe (Mm00504869_m1) or 0.5 μL human probe (Hs00174717_m1), and 5 μL LightCycler 480 probe master mix (Roche, cat no. 04887301001) per well in a 384-well plate (Roche, cat no. 04887301001). Real-time PCR was done in a LightCycler 480 Real-Time PCR system (Roche). Each siRNA was tested at least two times and data were normalized to cells transfected with a non-targeting control siRNA. To calculate relative fold change, real time data were analyzed using the ΔΔCt method and normalized to assays performed with cells transfected with a non-targeting control siRNA.

### *In vivo* siRNA screen

C57BL/6J mice at approximately eight weeks of age were s.c. injected with siRNA formulated in 1× PBS (pH 7.2). Animals were euthanized, and livers harvested and snap frozen in 15 mL polycarbonate sample collection jars (Thermo Fisher Scientific, 2116-0015) in liquid nitrogen, 7 and 21 days post-injection; each jar contained two steel balls (SPEX SamplePrep 2155) to assist with grinding. Frozen livers were ground in GenoGrinder 2010, and liver lysates were prepared for mRNA quantitation. RNA was extracted using PureLink Pro 96 total RNA Purification Kit. Total RNA was reverse transcribed using ABI High Capacity cDNA Reverse Transcription Kit, as mentioned previously. Quantitative PCR was performed as mentioned previously, using the Roche LightCycler 480 Probes Master Mix in the Roche 480 LightCycler Real Time PCR System (Roche) to determine the relative abundance of *CD81* (TaqMan probe Mm00504869_m1) mRNA and GAPDH (Applied Biosystems, 4352339E) as the housekeeping gene.

### Flow cytometry

Fluorescence staining and analysis was performed as previously described (Novobrantseva and Oza). Fc Block (2.4G2) and anti-mouse antibodies against CD45 (30F11) PerCP-Cy5.5, CD19 (1D3) SuperBright780, CD11b (M1/70) fluorescein isothiocyanate, F4/80 (T45-2342) Brilliant Violet 480 were purchased from BD Biosciences or Thermo Fisher Scientific. Stained cells were analyzed using BD FACSSymphony A3 (BD Biosciences). Analysis was done using FlowJo software (Flowjo) and data was graphed, and t test analyses were calculated in GraphPad Prism v.10 (GraphPad).

### Mosquito rearing and sporozoite production

*Anopheles stephensi* mosquitoes were reared at the Center for Global Infectious Disease Research, Seattle Children’s Research Institute (12-h light/dark cycle, 27°C, and 75% humidity) before infection with *P. yoelii* (Py) or *P. falciparum* (Pf), parasites constitutively expressing a GFP-luciferase fusion protein as previously described.^21^^,^^25^^,^^36^ Mosquitoes were provided 8% dextrose in water containing para-amino benzoic acid.

To induce the formation of *Py*GFPLuc gametocytes, female 6–8-week-old Swiss Webster mice were injected intraperitoneally with cryopreserved blood stage parasites. Production of *Pf*GFPLuc gametocytes followed established *in vitro* methodology. Asexual and sexual *Pf*GFPLuc cultures were maintained in RPMI 1640 containing 25 mM HEPES, 2 mM L-glutamine, 50 μM hypoxanthine, and 10% A+ human serum using O+ erythrocytes under an atmosphere of 5% CO_2_, 5% O_2_, and 90% N_2_ at 37°C. Mixed gametocyte cultures were initiated at 1% parasitemia in 5% hematocrit and maintained for 14–17 days with daily media changes. For both *Py* and *Pf*, the presence of mature gametocytes was confirmed by observation of exflagellation by light microscopy before feeding to female *Anopheles stephensi* mosquitoes 3–7 days post-emergence. Following blood feeding, mosquitoes were maintained at a temperature appropriate for the maximum development of the parasite (24°C for Py and 27°C for Pf). Midguts were dissected from blood-fed mosquitoes between day 7–10 post-feed to determine the percentage of infected mosquitoes as indicated by the presence of oocysts. Infection of mosquitoes was confirmed 14 days post-blood feed by dissecting the salivary glands from a small number of mosquitoes to confirm the presence of transmissible sporozoites.

### Mosquito bite challenge

C57Bl/6 or FRG-HuHep mice were challenged by infectious *Py*GFPLuc of *Pf*GFPLuc mosquito bites, and the resulting liver infection measured using bioluminescence as previously described.^37^^,^^38^ For *Py*GFPLuc infections, the number of female mosquitoes in each cup was normalized such that each cup contained 20 infectious mosquitoes (for example, if 91% of mosquitoes were infected based on oocyst counts then each cup contained 22 mosquitoes). C57Bl/6 mice were anesthetized with ketamine/xylazine and presented to the cups for 15 min with rotation of mice between cups every minute to normalize the force of infection between all mice and encourage mosquito probing behavior. For PfGFPLuc infections, cartons containing female mosquitoes was prepared from a mosquito batch with >50% infection and an average of >10 oocysts per mosquito midgut. The number of mosquitoes in each carton was determined by the number of mice to be infected, with ∼50 mosquitoes per mouse placed in each carton up to a maximum of 250 mosquitoes (sufficient for 5 mice to be infected). FRG-HuHep mice, in groups of up to five, were anesthetized with isoflurane via a nosecone and presented to the mosquito-containing carton for 10 min, with lifting of mice every minute to encourage mosquito probing behavior. Following anesthesia (ketamine/xylazine or isoflurane), mice were returned to their home cage and monitored for return to normal activity.

### Measurement of infection

The efficacy of siRNAs to block infection was measured as reduction in liver infection as well as protection from blood infection. Bioluminescence of the luciferase reporter was used to measure liver infection in mice at the end of liver stage development (44 h post-infection for *Py*GFPLuc, and 6 days post-infection for *Pf*GFPLuc). Briefly, the abdomens of all mice were shaved to remove their pigmented fur before injection of 150 μL of D-luciferin (GoldBio, St. Louis, USA). Mice were anesthetized with isoflurane, and then the bioluminescence emanating from the liver region was measured using an *In Vivo* Imaging System (IVIS, PerkinElmer). The total luminescence was converted to percentage of control (using a CD81 scramble siRNA, or naive mice as indicated) for presentation purposes. For experiments with PyGFPLuc, the measurement of liver infection was supplemented with a sensitive assessment for the presence of any viable liver parasites below the limit of detection by IVIS. Thin blood smears were collected from mice 3–14 days post-infection, fixed with 100% methanol and stained with Giemsa (Sigma-Aldrich, St Louis, USA). The first day that blood parasites were detected on Giemsa-stained thin blood smears was recorded and the delay to this observation (“patency”) recorded relative to control mice.

### Statistical analyses

All analyses were performed using GraphPad Prism 9 for macOS. Non-parametric tests were conducted where variance was unequal between groups. Differences were considered statistically significant when *p <* 0.05.

## Data availability

Data will be available upon request after publication.

## Acknowledgments

This work was conducted under Sponsored Research Agreement with Alnylam Pharmaceuticals. Experimental animal work was funded through internal research funds of SHI Kappe. We thank the former Center for Infectious Diseases Research insectary staff, principally Heather Kain for mosquito rearing and production. We additionally thank the former Center for Infectious Diseases Research vivarium staff for rodent maintenance. We also thank Alnylam’s siRNA synthesis team for expert technical assistance. The graphical abstract was generated by using BioRender (www.biorender.com).

## Author contributions

S.R.W.J. and K.S.H.I. conceived the study. S.R.W.J. performed the *in vivo* experiment for malaria infections. S.R.W.J., K.S.H.I., S.A., F.E., and H.P. wrote the paper. Y.K., M.S., N.T., H.P., and F.E. performed the experiment and analysis to validate the siRNAs.

## Declaration of interests

The authors declare no competing interests.

## Supplemental information

Document S1. Figure S1

Table S1. List of the mRNA sequences targeted by the CD81 siRNAs in the *in vitro* screen The table also shows fraction of transcript remaining in Hep3B cells and mouse primary hepatocytes at various siRNA concentrations, as measured by qPCR (data plotted in Figure 1).

Document S2. Article plus supplemental information
