# Supplementary material for: Protection from malaria infection using liver-targeted siRNA
Source: Mol Ther Methods Clin Dev. 2025 Jun 18;33(3):101516. doi: 10.1016/j.omtm.2025.101516 (PMC12269589; doi:10.1016/j.omtm.2025.101516)
Supplement: Document S1. Figure S1 [file mmc1.pdf]

OMTM, Volume *33*

Supplemental information

Protection from malaria infection using liver-targeted siRNA

R.W.J. Steel, A. Schepis, T. Nguyen, S. Milstein, K. Yucius, H.C. Tu, E. Fishilevich, P. Haslett, and S.H.I. Kappe

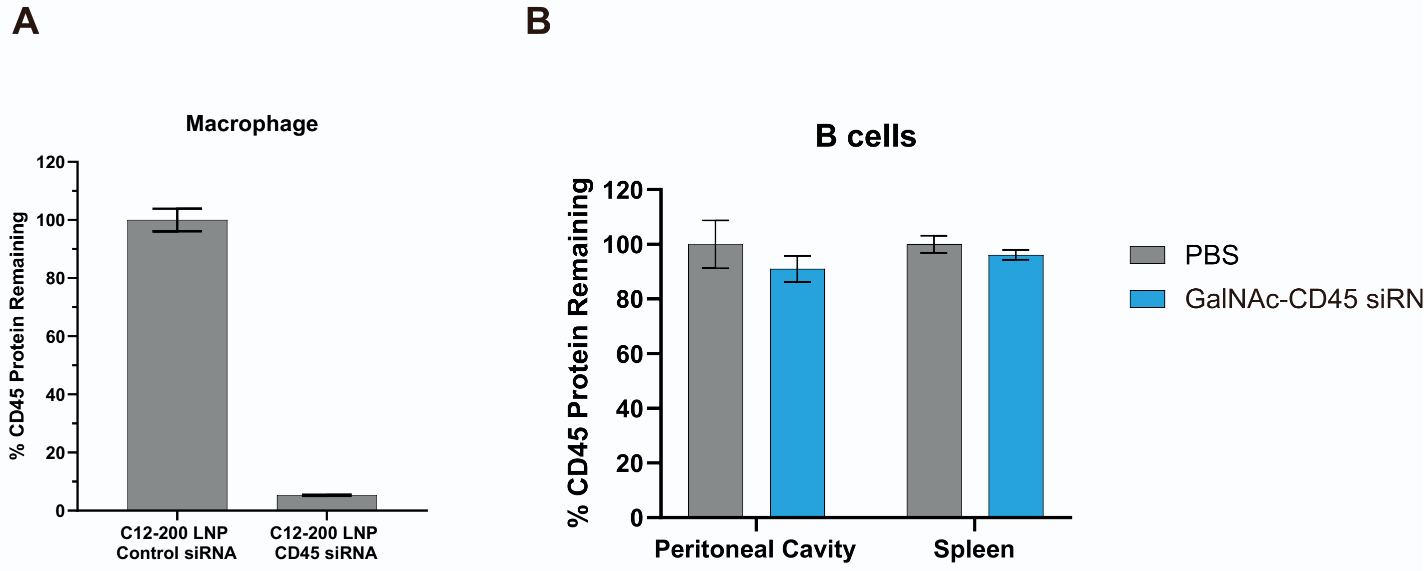

Figure S1. GalNAc-conjugated CD45 siRNA does not mediate target suppression in B cells. A. The efficacy of CD45 siRNA sequence was validated via LNP delivery to macrophages. C12-200 Lipid nanoparticles (LNP) containing either siRNA targeting CD45 (unconjugated) or non-targeting siRNA were intravenously injected into C57BL/6 mice (n=3/group) at a dose of 1 mg/kg. After 72 hours peritoneal cavity cells were collected, and large peritoneal macrophages were analyzed by flow cytometry for CD45 protein inhibition. CD45 median fluorescence intensity (MFI) was normalized to mean C12-200 LNP control siRNA group MFI. Silencing was statistically significant $p<0.0001$). **B.** GalNAc-conjugated siRNA targeting CD45 (same sequence as in **A**) or phosphate buffered saline (PBS) were subcutaneously injected into C57BL/6 mice (n=3/group) at a dose of 30 mg/kg. After 7 days peritoneal cavity cells and spleen were collected and B cells were analyzed by flow cytometry for CD45 protein inhibition. CD45 median fluorescence intensity (MFI) was normalized to mean PBS group. No statistical significance observed for spleen (p=0.14) or peritoneal cavity (p=0.19).

Table S1. List of the mRNA sequences targeted by the CD81 siRNAs in the *in vitro* screen. The table also shows fraction of transcript remaining in Hep3B cells and mouse primary hepatocytes at various siRNA concentrations, as measured by qPCR (data plotted in Figure 1).
